# Animal Welfare Control—Inspection Findings and the Threshold for Requesting a Police Investigation

**DOI:** 10.3389/fvets.2021.736084

**Published:** 2021-09-23

**Authors:** Elli Valtonen, Tarja Koskela, Anna Valros, Laura Hänninen

**Affiliations:** ^1^Department of Production Animal Medicine, Research Centre for Animal Welfare, University of Helsinki, Helsinki, Finland; ^2^Department of Law, Faculty of Social Sciences and Business Studies, University of Eastern Finland, Joensuu, Finland

**Keywords:** investigation requests, non-compliance, companion animals, animal welfare, inspections

## Abstract

An increasing proportion of animal welfare violations in Finland are related to companion animals. However, only a small number of these issues are investigated or prosecuted. The aims of this study were (i) to describe the inspection findings and the resulting actions of the official municipal veterinarians in the Finnish Capital Region and (ii) to identify the factors that predict their submissions of investigation requests to the police. Our data consisted of animal welfare complaints and official veterinarians' inspection reports and decisions from 811 cases of animal welfare control in the Finnish Capital Region. The data covered the period from March 2019 to April 2020. We performed logistic regression analyses to identify the factors that best predict when official veterinarians detect non-compliances and report the cases for police investigation. In 86% (696/811) of the cases, the veterinarians performed at least one animal welfare inspection, and/or received information from the police, or otherwise investigated the complaint. The most common forms of non-compliance were lack of basic maintenance and care (42%, 295/696) and insufficient veterinary care (27%, 185/696). The veterinarians requested for a police investigation in 9.6% (44/460) of all cases with detected non-compliances. The best predictors for detecting non-compliances with the animal welfare legislation were complaints of insufficient veterinary care (OR 1.9, CI 1.1–3.4), the cases assessed by the information from the police and/or an animal shelter (OR 15.2, CI 7.9–29.2), at least one inspection in an animal's premises with prior warning (OR 11.2, CI 5.5–22.6), and without prior warning (OR 17.0, CI 9.7–29.5). Complaints of violence against animals were negatively associated with detecting non-compliances (OR 0.5, CI 0.3–0.9). However, the detection of violence against animals predicted requests for police investigations (OR 9.3, CI 3.1–27.9), as did the execution of permanent urgent measures by official veterinarians (OR 4.9, CI 1.9–12.9). To improve the animal welfare control system and the investigation of crimes against animals, cooperation between officials should be developed. Further studies are needed to improve the understanding of the prevalence of violence against animals, and to advance methods used by animal welfare control to identify cases of violence.

## Introduction

Alongside increasing urbanization, the popularity of companion animals has risen. In Finland, between 2012 and 2016, the number of households with a dog increased by 13.4% and the number of households with a cat by 4.2%; almost one third of all households owned a companion animal (1). Consequently, there has also been an increase in the number of animal welfare problems that involve companion animals. Moreover, the observed maltreatment of companion animals often leads to action: in 2020, more than 80% of the urgent enforcement measures and nearly 60% of the orders and prohibitions executed by animal welfare authorities in Finland were for companion animals (2). Prior research has indicated that small vertebrates kept as companion animals are common victims of intentional violence (3, 4); however, only a small proportion of these crimes are investigated or prosecuted (5, 6).

In Finland, animal welfare supervision and control are regulated primarily by the Animal Welfare Act (AWA 247/1996)^1^. The Act states that animals must be treated well, no unnecessary distress may be caused to them and inflicting unnecessary pain and distress on animals is prohibited. In addition, maintaining the health of animals must be promoted when keeping them, and the physiological and behavioral needs of the animals must be taken into account. Further provisions are issued by the Animal Welfare Decree (396/1996)^2^ and the decrees of the Council of State and Ministry of Agriculture and Forestry. For companion animals, the Decree of the Council of State (674/2010)^3^ lays down more detailed standards for minimum welfare requirements. At the municipal level, animal welfare complaints must be “sufficiently and appropriately examined, by acquiring the information and evidence necessary for a decision to be made on the matter”^4^; these assessments are carried out by the municipal veterinarian, the municipal health protection control officers, or the police^1^. Specific offices were established for animal welfare control in Finland in 2009 (7), and official veterinarians currently oversee most of the complaints and perform the necessary examinations. The authorities have the right to inspect an animal and its premises. They may also provide the owner or possessor of the animal with advice, orders, or prohibitions, and they can institute urgent measures to acquire alternative care for the animal, or have the animal sold or killed^1^. Previous research has been critical of official veterinarians and their passive approach to initiating and executing necessary enforcement measures (8, 9). However, there is a current lack of research examining the content of animal welfare complaints and the features of cases that result in administrative measures.

Previous studies on official animal welfare control and criminal sanctions in Finland (9–13) have mainly focused on production animals and have generally recorded passive forms of maltreatment; forms of active violence are rarely detected during inspections (10) and most infrequently prosecuted in the courts (11). Only the most severe cases with a prolonged lack of adequate premises, feeding and watering as the main non-compliances seem to result in a criminal procedure (11), and the features of these cases have not been clearly identified.

Official veterinarians must notify the police of any violations of the animal welfare legislation^1^. However, based on the number of animal welfare inspections^5^ and the recorded animal welfare offenses (14), most violations of the animal welfare legislation are not officially reported to the police, and those violations that are reported are not investigated as often as the average of all crimes. Previous research on animal welfare procedures for production animals has emphasized the role of veterinarians in criminal procedures (8, 9, 11) and identified that veterinarians' readiness to request for police investigations is relatively low (8, 9). However, the studies have not addressed the cases relating to companion animals, or the features of the cases that lead to investigation requests by official veterinarians.

The aims of this study were (i) to describe the inspection findings and the resulting actions of the official municipal veterinarians in the Finnish Capital Region and the measures taken to manage animal welfare offences, and (ii) to identify the factors that predict their submissions of investigation requests to the police. This research will help improve the animal welfare control system and the investigations of crimes against animals.

## Materials and Methods

### Documents of Animal Welfare Control at the Municipal Level

Our raw data consisted of complaints, inspection reports and decisions concerning non-compliances with the animal welfare legislation. The following list of cases were excluded from the data set: the complaints concerning zoos or permanent animal shows that are controlled by the Regional State Administrative Agencies; wild animals in need of rescue or crimes against the Nature Conservation Act^6^, as these inspections are not generally performed by the municipal authorities; the complaints (<10) concerning animals kept for production purposes; the inspections of pet shops and stables that were conducted according to a yearly plan and without suspicion of non-compliances with the animal welfare legislation; and cases of lost and found animals that were not linked to violations of the animal welfare legislation. Overall, the data set comprised of 811 cases that were reported between 1 March 2019 and 30 April 2020 in the Finnish Capital Region. Some cases included more than one incident, i.e., several complaints, inspections and/or decisions during the study period.

The municipal authorities received complaints that varied in detail and length either by e-mail or by phone. To accept the complaints for consideration, the municipal units required certain essential information; specification of the animal species, a description of the suspected non-compliance with the animal welfare legislation, and the full address of the animal's location. As a minimum, the inspection reports and decisions contained the following details: the name and contact details of the authority performing the inspection, the name and full address of the owner or possessor of the animal, the names or statuses of the people present during the inspection, the species and number of animals inspected, and the essential observations of the inspector. If the official veterinarian detected a non-compliance with the animal welfare legislation, the documents contained advice, prohibitions, orders and/or urgent measures, and justifications for the actions taken. The hearing of the owner or possessor was documented for prohibitions and orders. For the urgent measures taken by the authority, the hearing was documented if carried out.

### Data Collection

The following data were collected for the analyses: (1) the animal species reported in the complaint (dogs, cats, other companion animal species, or wild animals; number of species); (2) the source of the complaint (information on animals still in their premises, or information from the police or animal rescuers regarding animals removed from their premises and transported to an animal shelter); (3) the reason for the complaint (Table 1); (4) the methods for investigating animal welfare (an inspection with or without prior warning; assessment by information received from an animal shelter or the police, or an investigation by phone or e-mail only); (5) the official veterinarians' observations of the animal species (dogs, cats, other companion animals, or wild animals; number of species) and non-compliances with the animal welfare legislation (Table 1); (6) the measures executed by the official veterinarians (advice (based on AWA 40 §), prohibitions and orders (AWA 42 §), temporary urgent measures (AWA 44 §), or permanent urgent measures (AWA 44 §), and requests for police investigations (AWA 63 §). Also, the number of incidents (inspections/police measures) per case (one/several) and whether the police provided executive assistance or otherwise participated in the animal welfare inspection (yes/no) were recorded.

**Table 1:** Non-compliances with the animal welfare legislation and other problems concerning animal welfare in the complaints received by the municipal animal welfare authorities.

**Violations of the animal welfare legislation (1.-7.) or other complaints (8.-11.) concerning animals**	**Description**
1. Inadequate nutrition	Inappropriate food or drink; lack of nutrition; excessive feeding
2. Inappropriate premises	Lack of space; dangerous objects, e.g., needles/ glass shards; animal escapes repeatedly; urine/feces on the floor/in the cage; feeding equipment not clean; inappropriate lighting; low air quality; lack of sufficient shelter against adverse weather conditions or excessive cold, heat, or humidity
3. Insufficient exercise (dogs)	Lack of exercise; no opportunity to urinate and defecate outdoors
4. Insufficient basic care	E.g., overgrown claws or tangled fur that cause pain and difficulties with movement; lack of other necessary care, e.g., washing or cleaning; too frequent breeding of female animals; too early weaning; welfare and health not monitored with adequate frequency
5. Insufficient veterinary care	Lack of veterinary care or euthanasia for a sick or injured animal
6. Abuse/violence	Kicking, hitting, or other physical violence; excessive exertion, unreasonable discipline and training; excessively rough handling; unnecessary frightening actions or agitation
7. Abandoned/overall neglect	Animals left without care; animals not reclaimed from animal shelter by their owner
8. Smell	Bad smell in the animal's premises; the animal smells strongly
9. Noise	Distracting/worrying noise of an animal
10. Aggressive animal	Aggression toward humans or other animals
11. Owner/possessor hospitalized, arrested, or deceased	Owner cannot temporarily or permanently arrange the care of the animal

*The definitions of non-compliances (1.-7.) also apply to the non-compliances detected during the inspections or other investigations*.

### Statistical Analyses

For the statistical analyses, we initially created five different variables to group the strongly related variables and to identify the relevant types of complaints; (1) abandoned animals; (2) violence toward an animal that was not an abandoned animal; (3) insufficient veterinary care that did not involve violence or an abandoned animal; (4) notifications of basic maintenance and care only (inappropriate premises, insufficient exercise of dogs, inadequate nutrition and insufficient basic care); or (5) complaints of noise, smell or aggressive animals or of owner being hospitalized, arrested, or deceased, with no complaints regarding animal welfare law violations.

In the next phase, the detected non-compliances were re-scored as: (1) abandoned animals; (2) at least one incident of violence toward an animal that was not an abandoned animal; (3) at least one incident of insufficient veterinary care that did not involve violence or an abandoned animal; or (4) basic maintenance and care only (inappropriate premises, insufficient exercise of dogs, inadequate nutrition and insufficient basic care). Many cases fell into more than one category concerning the method of the investigation; therefore, they were also scored according to the investigative measures performed: (1) inspection(s) performed without prior warning at least once; (2) inspection(s) performed with prior warning; (3) no inspection, cases concerning animals removed to an animal shelter, assessed using information from the police or a shelter; and (4) no inspection, investigation only by phone or e-mail (e.g., by discussing with an owner, and/or assessing the animal's health or premises by photographs or clinical reports). Additionally, we scored the stringency of the measures executed by the official veterinarians: (1) no measures executed, or written advice provided at least once, but no orders or prohibitions given or urgent measures executed; (2) orders or prohibitions given at least once, but no urgent measures executed; (3) temporary urgent measures executed at least once, no permanent urgent measures; and (4) permanent urgent measures executed at least once. We used chi-square tests and Fisher's exact test or Fisher-Freeman-Halton exact test to analyze the following associations: (1) between all the potential explanatory variables and the outcome variables of interest (see Supplementary Materials 1, 2); and (2) between the animal species and the non-compliances reported in the complaints or detected by the veterinarians.

Based on the univariate analyses of the relevant explanatory variables, we formed two separate logistic regression models to examine which factors best predicted (1) the official veterinarians' detection of non-compliances (Model 1) and (2) their reporting of cases for police investigation (Model 2). The explanatory variables with Fisher's exact test or Fisher-Freeman-Halton exact test *p* < 0.2 were considered potential for the logistic models. For Model 1, the following variables were considered: the animal species reported in the complaint; the non-compliance type reported in the complaint; the source of the complaint; the method of investigation; more than one inspection/police measure, and the animal species detected. For Model 2, we considered the following variables: a dog or a cat detected; the type of detected non-compliance; the method of investigation; the stringency of executed measures, and more than one inspection/police measure.

Relationships between relevant explanatory variables were examined by pair-wise associations using chi-square tests and Fisher's exact tests. As the non-compliance types reported in the complaints were associated with the animal species, the animal species categories were excluded from the first model. Similarly, cases with veterinarians' detection of more than one species were nearly always those with inspection(s) performed in animal premises, and thus the category of “several species detected” was excluded from the first model. Furthermore, the cases with several incidents were, by definition, those with animals removed from their premises and/or inspections performed in the animal premises, and, in addition, associated with the more stringent measures performed by the veterinarians. Thus, the category of “more than one inspection/police measure” was excluded from both regression models. Also, as the cases of animals removed to a shelter by the police were most frequently investigated on basis of the information provided by the police or a shelter, the variable “source of complaint” was excluded from the first model. All relevant interactions were tested, but no significant associations with the outcome variables were found.

As a result, the following models were formed: to explore the official veterinarians' detections of law violations in investigated cases (Model 1, any violation/ no violation, *N* = 696), the main effects included in the model were the complaint type and the method of the investigation; and to examine the requests for police investigation in cases with detected non-compliances with the legislation (Model 2, at least one investigation request submitted by the official veterinarian/ no investigation request submitted, *N* = 460), the model included the type of detected non-compliances and the stringency of the executed measures. Both models were evaluated with Hosmer and Lemeshow tests of goodness of fit and ROC curve.

Statistical analyses were performed with IBM SPSS Statistics for Windows Version 27.0 (IBM Corp., Armonk, NY, USA). Statistical significance was accepted at a confidence level of 95% (*p* < 0.05).

## Results

### Sources of the Complaints

In 75.5% (612/811) of the cases, the complaints were related to animals in their premises. In these cases, the information source was most frequently a member of the public, but also other stakeholders, such as health care services, social services and landlords, made complaints. However, the status of the information source was not always known, as also anonymous complaints were accepted for consideration.

In 24.5% (199/811) of the cases, the animals were urgently removed from their premises at least once, and transported to an animal shelter by the police, or, in some cases, by the animal rescue units of the municipal rescue departments, the Helsinki Humane Society HESY or another party. In acute situations, either the police or the official veterinarian executed the immediate urgent measures, i.e., administered a decision of providing care for the animal or euthanizing it. To facilitate further action, the police and/or an animal shelter provided the municipal official veterinarians with the animal's necessary health and owner information.

### Types of Non-compliances Reported to the Official Veterinarians

Overall, 70.0% (568/811) of the complaints concerned dogs, 27.0% (219/811) cats, and 9.6% (78/811) other companion animals. The category of “other companion animals” included horses, sheep, guinea pigs, hamsters, rabbits, rats, gerbils, hedgehogs, snakes, geckos, turtles, tortoises, fish, parrots, chickens, spiders, insects, and snails. Only 0.5% (4/811) of all cases concerned wild animals. In 6.8% (55/811) of the cases, the complaints included more than one species. The non-compliances that were reported most frequently related to the basic maintenance and care of the animals (46.5%, 377/811); within this category, inappropriate animal premises (46.1%, 374/811) and insufficient exercise for dogs (25.5%, 207/811) were most frequently reported. These were followed by complaints of insufficient veterinary care (20.1%, 163/811). For all frequencies, see Table 2.

**Table 2:** Frequencies of reported, investigated, and detected non-compliances and related administrative measures.

**Non-compliance or disturbance reported in the complaint, main category**	**Non-compliance or disturbance reported in the complaint, subcategory**	**% (*N*)^**a**^**	**Inspection or other investigation performed by an official veterinarian, % (*N*)^**b**^**	**Non-compliance detected, % (*N*)^**c**^**	**No measures, % (*N*)^**e**^**	**Advice only, % (*N*)^**e**^**	**Orders/ prohibitions, no urgent measures, % (*N*)^**e**^**	**Urgent measures, % (*N*)^**e**^**
All cases		100 (811)	85.8 (696)	66.1 (460)^d^	7.4 (34)^d^	27.8 (128)^d^	25.7 (118)^d^	39.1 (180)^d^
Insufficient basic maintenance and care		65.1 (528)	85.4 (451)	42.4 (295)	7.1 (21)	32.2 (95)	29.5 (87)	31.2 (92)
	Inappropriate premises	46.1 (374)	89.3 (334)	32.6 (227)	6.2 (14)	30.8 (70)	27.3 (62)	35.7 (81)
	Insufficient exercise	25.5 (207)	81.2 (168)	7.5 (52)	1.9 (1)	34.6 (18)	25.0 (13)	38.5 (20)
	Inadequate nutrition	16.6 (135)	91.9 (124)	12.6 (88)	3.4 (3)	17.0 (15)	42.0 (37)	37.5 (33)
	Insufficient basic care	14.7 (119)	89.9 (107)	10.3 (72)	5.6 (4)	37.5 (27)	23.6 (17)	33.3 (24)
Insufficient veterinary care		20.1 (163)	96.3 (157)	26.6 (185)	6.5 (12)	16.8 (31)	34.6 (64)	42.2 (78)
Abuse/ violence		14.7 (119)	81.5 (97)	3.3 (23)	8.7 (2)	39.1 (9)	13.0 (3)	39.1 (9)
Abandoned/ overall neglect		10.7 (87)	98.9 (86)	12.8 (89)	3.4 (3)	0 (0)	1.1 (1)	95.5 (85)
Animal disturbing the environment only		3.8 (31)	38.7 (12)	16.1 (5)^d^	0	20.0 (1)	60.0 (3)	20.0 (1)
Owner hospitalized, arrested, or deceased		13.7 (111)	98.2 (109)	56.0 (61)^d^	18.9 (21)^f^	5.4 (6)^f^	4.5 (5)^f^	71.2 (79)^f^

*N = 811*.

a *Of all cases*.

b *Of cases with the non-compliance reported in the complaint*.

c *Of all cases investigated by an official veterinarian*.

d *Any non-compliance*.

e *Of cases with a detected non-compliance*.

f *Of cases with the owner hospitalized, arrested, or deceased*.

The complaints concerning insufficient veterinary care, abandoned animals, or a hospitalized, arrested, or deceased owner were recorded at the same frequency for dogs, cats, and other companion animals. Complaints of inappropriate nutrition [$X_{\left(1,\text{N}=811\right)}^{2}$ = 9.0, *p* = 0.003], inappropriate premises [$X_{\left(1,\text{N}=811\right)}^{2}$ = 12.4, *p* < 0.001], and lack of basic care [$X_{\left(1,\text{N}=811\right)}^{2}$ = 4.1, *p* = 0.05] were more frequently related to cats and “other companion animals” than dogs. Complaints of smell were more often related to cats [$X_{\left(1,\text{N}=811\right)}^{2}$ = 16.8, *p* < 0.001] and complaints of noise [$X_{\left(1,\text{N}=811\right)}^{2}$ = 38.0, *p* < 0.001], aggressive animals [$X_{\left(1,\text{N}=811\right)}^{2}$ = 23.4, *p* < 0.001], and violent treatment [$X_{\left(1,\text{N}=811\right)}^{2}$ = 20.0, *p* < 0.001] to dogs. Complaints of insufficient exercise were only recorded for dogs.

### Investigations and Detected Non-compliances

The municipal veterinarians investigated the complaints in 85.5% (696/811) of the cases. They performed at least one physical inspection in the animal premises in 50.7% (411/811) of the cases, received information for their decisions from an animal shelter or the police in 24.5% (199/811) of the cases, and inquired the case by phone calls or e-mails in 21.3% (173/811) of the cases. In 14.8% (103/696) of the investigated cases, there was more than one incident (inspections performed and/or measures taken) registered during the study period. The prevalence of cases with more than one incident was significantly higher for the cases with animals removed from their premises by the police than for the cases of animals in their premises (21.6% (43/199) and 15.1 (75/497), [$X_{\left(1,\text{N}=696\right)}^{2}$ = 4.3, *p* = 0.04]. The police gave executive assistance to the official veterinarian or otherwise participated in the inspections in 12.4% (51/411) of the inspected cases. In 8.6% (70/811) of all cases the official veterinarian visited the animal premises but was unable to complete any inspection.

Most complaints only reported one type of non-compliance with the animal welfare legislation (range 0–5, median 1). Similarly, the veterinarians typically observed one type of non-compliance (range 0–5, median 1) when performing an inspection or otherwise investigating a case. Overall, the veterinarians detected non-compliances in 66.1% (460/696) of all the examined cases. More precisely, they identified non-compliances in 77.7% (262/337) of the cases that they inspected at least once without prior warning, in 70.3% (52/74) of the cases that they inspected with prior warning, and in 72.1% (124/172) of the cases they assessed using information from the police or animal shelter. The frequency of detected non-compliances did not differ significantly between inspections with and without prior warning [$X_{\left(1,\text{N}=411\right)}^{2}$ = 1.9, *p* = 0.2]. Non-compliances were identified in 19.5% (22/113) of the cases that were only investigated by phone or e-mail. The most common non-compliances concerned basic maintenance and care (42.4%, 295/696); within this category, inadequate premises (32.6%, 227/696) were most frequently detected. This was followed by insufficient veterinary care (26.6%, 185/696) and abandoned or overall neglected animals (12.8%, 89/696). Violence toward an animal was detected in 3.3% (23/696) of the investigated cases (Table 2).

When inspections were physically performed on an animal's premises, dogs were detected in 67.6% (278/411), cats in 34.5% (142/411), and other species in 15.8% (65/411) of the cases. Species that were different from those reported in the complaint were observed in 5.2% (36/696) of all the investigated cases and 7.8% (32/411) of all the physically inspected cases. The unexpected observations of animals were most often of species other than dogs (72.2%, 26/36) and were associated with physical inspections conducted on the animal's premises; 88.9% (32/36) of these were detected by a veterinarian performing an inspection in an animal's premises instead of assessing information received from another party [$X_{\left(1,\text{N}=696\right)}^{2}$ = 14.0, *p* < 0.001].

### Measures Executed by the Official Veterinarians

In 7.4% (34/460) of all cases with detected non-compliances, the veterinarian did not perform any administrative measures, but only confirmed the animal's welfare by phone or e-mail. In 27.8% (128/460) of the cases, they gave written advice that was attached to an inspection report, but did not undertake any other measures. In 25.7% (118/460) of the cases, the veterinarian gave orders and/or prohibitions, but did not execute urgent measures. In 39.1% (180/460) of the cases, the authorities undertook urgent measures to provide the animal with care, rehome or euthanize the animal. The urgent measures became permanent in 25.7% (118/460) of the cases, i.e., the animals were rehomed or euthanized.

In 4.4% (36/811) of all cases, the animals were placed in a shelter by the police, and no non-compliance with the animal welfare legislation was detected by an official veterinarian. In 1.8% (15/811) of all cases, this resulted in permanent urgent measures executed by the veterinarian. These cases concerned either wild animals or companion animals with owners that were hospitalized, arrested, or deceased. Altogether, the police or an official veterinarian applied urgent measures at least once in 26.6% (216/811) of all cases. In 61.6% (133/216) of these cases, the urgent measures became permanent.

Urgent measures were implemented in 71.2% (79/111) of all cases with a hospitalized, arrested, or deceased owner. Furthermore, urgent measures were executed more frequently in cases that involved an abandoned or overall neglected animal [95.5%, $X_{\left(1,\text{N}=460\right)}^{2}$ = 147.2, *p* < 0.001] than in other cases with detected non-compliances. Additionally, either urgent measures or orders or prohibitions were more often implemented when insufficient veterinary care was detected [76.8%, 142/185, $X_{\left(1,\text{N}=460\right)}^{2}$ = 19.4, *p* < 0.001]. In contrast, in cases with insufficient basic maintenance and care, no measures were performed or the owner was provided only with advice more frequently than in other cases [39.3%, 116/295, $X_{\left(1,\text{N}=460\right)}^{2}$ = 6.1, *p* = 0.02]. For all frequencies, see Table 2.

### Predictors for the Detection of Non-compliances

Model 1 containing the non-compliance type reported in the complaint and the method of the investigation was statistically significant (*N* = 696, *X*^2^ = 165.5, *p* < 0.001). The area under the ROC curve was 77% (CI 73–81%, *p* < 0.001), and the *p*-value for the Hosmer and Lemeshow goodness of fit test was 0.7 (*X*^2^ = 4.3).

The strongest predictors for the detection of non-compliances were the cases involving animals removed to an animal shelter by the police and assessed by the information from the police or a shelter, and cases with at least one inspection with or without prior warning. In addition, complaints of insufficient veterinary care predicted the detection of non-compliances better than complaints of insufficient basic maintenance and care. However, complaints of violence and abuse, complaints of hospitalized, arrested, or deceased owners, and complaints of animals disturbing the environment were all negatively associated with the detection of non-compliances (Table 3).

**Table 3:** The results of the logistic regression analysis for the Model 1.

**Variable**	**Category**	***N***	**Cases with detected non-compliances**	**OR**	**95% CI**	***P*-value**
**Non-compliance type reported in the complaint**						<0.001
	Insufficient basic maintenance and care only	310	205	1 (Ref)		
	Owner hospitalized, arrested, or deceased, or animal disturbing the environment	66	28	0.25	0.13–0.47	<0.001
	Insufficient veterinary care	131	99	1.94	1.12–3.36	0.02
	Abuse/violence	95	53	0.54	0.32–0.91	0.02
	Abandoned/overall neglect	94	75	1.46	0.77–2.80	0.2
**Method of investigation**						
	Investigated by phone or e-mail only	113	22	1 (Ref)		
	Assessed by the information received from an animal shelter and/or the police	172	124	15.18	7.89–29.18	<0.001
	Inspection(s) in an animal's premises with prior warning	74	52	11.18	5.53–22.62	<0.001
	Inspection(s) in an animal's premises without prior warning	337	262	16.95	9.74–29.50	<0.001

*Predictors for veterinarians detecting non-compliances with the animal welfare legislation in the examined cases*.

*Non-compliances were detected in 66.1% (460/696) of the examined cases. N = 696*.

*OR, Odds ratio; CI, Confidence interval*.

### Requests for Police Investigation

The processes of notifying the police of non-compliances with the animal welfare legislation varied. The official municipal veterinarians requested for a police investigation or otherwise ensured that a criminal investigation was instituted in 9.6% (44/460) of all cases with detected non-compliances. In four cases, the official veterinarian did not detect a non-compliance but did initiate a police investigation that was based on the complaint.

In 14.6% (7/48) of the cases with an investigation request, the official veterinarian had given orders or prohibitions. In 16.7% (8/48) of the cases, temporary urgent measures were executed by the official veterinarian, and in 43.8% (21/48) of the cases, urgent measures were permanent. In three cases, an animal had been illegally imported. In two cases each, an animal died prior to any measures being taken, an owner arranged for an animal to be euthanized, an owner gave their animal away, and an animal was seized by the police. In one case the police had ensured that the animal was removed from the owner and relocated to appropriate premises.

Model 2 containing the non-compliance type detected by the official veterinarian and the stringency of the executed measures was statistically significant (*N* = 460, *X*^2^ = 31.5, *p* < 0.001). The area under the ROC curve was 71% (CI 62–79%, *p* < 0.001), and the *p*-value for the Hosmer and Lemeshow goodness of fit test was 0.98 (*X*^2^ = 1.5). The logistic regression model showed that the detection of violence against animals or the execution of permanent urgent measures by official veterinarians predicted requests for police investigations (Table 4).

**Table 4:** The results of the logistic regression analysis for the Model 2.

**Variable**	**Category**	***N***	**Investigation request submitted to the police**	**OR**	**95% CI**	***P*-value**
**Non-compliance type detected**						<0.001
	Insufficient basic maintenance and care only	190	11	1 (Ref)		
	Insufficient veterinary care	159	14	1.14	0.48–2.71	0.8
	Abuse/ violence	22	9	9.27	3.07–27.94	<0.001
	Abandoned/ overall neglect	89	10	0.75	0.27–2.09	0.5
**Executed measures**						0.006
	No measures or only advice	162	9	1 (Ref)		
	Orders and prohibitions	118	7	1.28	0.44–3.75	0.6
	Urgent measures, temporary	62	7	3.07	0.99–9.49	0.05
	Urgent measures, permanent	118	21	4.88	1.85–12.87	0.001

*Predictors for veterinarians requesting for a police investigation in cases with detected non-compliances. Investigation request was submitted in 9.6% (44/460) of the cases with detected non-compliances. N = 460*.

*OR, Odds ratio; CI, Confidence interval*.

## Discussion

A lack of basic maintenance and care was the most common issue reported in the animal welfare complaints and the most frequent non-compliance with the animal welfare legislation detected by the official municipal veterinarians in the Finnish Capital Region. The best predictors for the official veterinarians detecting non-compliances with the animal welfare legislation were as follows: complaints of insufficient veterinary care, the authorities performing inspections, or assessing the cases of animals removed to an animal shelter using the information from the police or a shelter. In contrast, complaints of violence against animals were negatively associated with the detection of non-compliances. However, the *detection* of violence against animals predicted requests for police investigations, as did the execution of permanent urgent measures by official veterinarians.

Overall, official veterinarians detected non-compliances with the animal welfare legislation in 66% of the cases they investigated. The most common concern, expressed in nearly half of all the animal welfare complaints, were deficiencies in basic maintenance and care of the animals; these complaints concerned inappropriate animal premises, nutrition, or basic care, or dogs with insufficient exercise or a lack of access to the outdoors for defecation. These were also the most frequently recorded forms of non-compliances and were observed in over 40% of the examined cases. This result is consistent with previous research on production animal welfare control. Studies have identified that inadequate lying areas for adult cattle, deficient housing conditions for calves, and a lack of enrichment material for pigs are the most frequent forms of non-compliance (10). In addition, dirty premises and inadequate feeding and watering have been the most common violations sanctioned in the courts (11). The results also showed that insufficient veterinary care was an issue in 20% of the complaints, and the veterinarians observed this non-compliance in more than a quarter of all examined cases. Approximately 25% of the observed non-compliances resulted in advice and another 25% led to orders and prohibitions. Urgent measures were executed in 40% of the cases of non-compliance, and two thirds of these cases resulted in the permanent rehoming or euthanizing of the animals.

We found that the best predictors for detecting non-compliances with animal welfare legislation were the official veterinarians performing at least one inspection or assessing the cases by the information from the police or an animal shelter. As often as possible, animal welfare inspections are conducted without prior warning, as notifying the owner or possessor frequently jeopardizes the purpose and the intended outcome of the inspection^4^. However, some cases in this study were inspected with prior warning or they were inquired by phone or e-mail. Based on authors' own experience, these measures may be applicable to certain cases, for example, when investigating animals that have been examined by a clinical veterinarian, or re-inspecting animals that have recently been seen in their premises by an official veterinarian. The official veterinarians detected violations of animal welfare legislation equally often on inspections with and without prior warning. This is in line with the results of Väärikkälä et al. (11, 15) indicating that the minimum requirements of animal welfare legislation are not clear to the owners or possessors of the animals, or that they are not able or motivated to follow them. Furthermore, when a veterinarian performed an inspection on an animal's premises, species not reported in the initial complaint were sometimes also observed. Cats and other small-sized companion animals are not as easy to see or hear as dogs; however, as animal welfare legislation applies for all animals^1^, it is of equal importance that the physical condition and premises of all animal species are assessed in suspected cases of non-compliance with the legislation. We argue that this further emphasizes that physical inspections on an animal's premises are essential.

Violence toward an animal was reported in 15% of the complaints but only detected in 3% of all cases, most rarely when compared to other categories of non-compliance. Also, complaints of violence were negatively associated with the official veterinarians detecting any non-compliances. These results are in line with previous research on production animals with active violence infrequently detected during inspections (10). It is likely that confirming cases of violence is challenging: animals can only be superficially examined during an inspection, and it is often impossible to prove the cause and mechanism of observed injuries without forensic pathology (16). Furthermore, although fearful and aggressive behavior is typical for abused dogs (17), exclusive signs of violence cannot be detected in animals' behavior. Thus, eyewitness accounts are crucial when confirming cases of animal abuse, and police investigations are necessary to record statements. Additionally, the possibility of false complaints of violence cannot be ruled out for some of these cases. To investigate the features and prevalence of violence against companion animals, a more comprehensive data set with investigated cases of violent offenses would be necessary.

The official veterinarians made investigation requests or otherwise ensured that the criminal procedures were initiated in 10% of all cases with detected non-compliances. There was variation in these practices and, in some cases, an inspection report was delivered to the police without a specific investigation request. It is possible that our data is not comprehensive: additional requests may have been made after the data collection period or criminal procedures may have been initiated through other channels. For example, a clinical veterinarian can report a case to the police, and an eyewitness of violence against an animal may be encouraged to make a direct investigation request, and the criminal procedure may proceed without a contribution from an official veterinarian. However, considering the legal requirement to notify the police of all suspected violations of the animal welfare legislation^1^, the number of investigation requests is small. Our results align with previous research by Wahlberg (9) and Koskela (8) that indicated that official veterinarians are relatively passive in terms of submitting investigation requests. The potential reasons for this reduced response include stress and fatigue due to excessive workloads and recurrent threatening situations (18), ambiguous definitions of unnecessary suffering (19), and reserving the limited resources of both the police and official veterinarians for the most serious cases. Also, compassion toward an animal owner and moral distress from reporting underprivileged people to the police may prevent an official animal welfare authority from initiating a criminal procedure (20, 21).

Our results highlight the role of the police in animal welfare control; in particular, their involvement in recognizing and transporting the severely neglected or abandoned animals to shelters. Official veterinarians were more likely to execute urgent measures when a case involved police intervention and transportation of an animal to an animal shelter. In addition, the police perform essential roles when seizing animals, investigating suspected crimes, and providing executive assistance or participating in animal welfare inspections (22). Also, recognition of violence against companion animals as a form of domestic violence (23, 24) further underlines the need for collaboration between police and official veterinarians. An investigation team for animal crimes was established in the Helsinki Police Department in 2018. This initiative should improve the quality and quantity of investigations of animal crimes and enhance the collaboration between the police and the official municipal veterinarians (22).

The inappropriate premises of companion animals, which was the most common type of non-compliance reported in our data, indicate a risk of unhealthy living conditions of the owners. Thus, our results support previous studies underlining the need for active cooperation between animal welfare control, social work, and health care services (25, 26). Current knowledge of the intertwined welfare of human and non-human animals (27, 28) and the explanations presented by people accused of crimes against animals [e.g., (4, 11)] indicate that violations of animal welfare reflect the economic hardship and untreated mental health issues of the animal owners. Studies from Canada and the Netherlands show that the animal welfare officers are aware of their role in the net of One Welfare and the essentiality of the health and social conditions of people to the health and welfare of their animals (20, 21).

We argue that, to a significant extent, the official veterinarians' threshold for making investigation requests sets the definition of unnecessary suffering. The key roles of veterinarians as initiators and expert witnesses in criminal procedures have been widely recognized, and the need for efficient cooperation between the official veterinarians and the police has been emphasized (8, 11, 29). The criminal procedure and the administrative process of an official veterinarian investigating the case often proceed in parallel, which allows cooperation. Our study supports the proposals of Väärikkälä et al. (11) and Koskela (22) who suggested that a joint education program for official veterinarians and the police would develop a shared understanding of unnecessary suffering as a definite condition of animal crime. We also recommend that a nationwide directive is introduced for official veterinarians concerning the minimum requirements for making an investigation request or otherwise notifying the police of violations of the animal welfare legislation.

## Conclusions

Insufficient basic maintenance and care of companion animals was the most common form of non-compliance with the animal welfare legislation; this was followed by insufficient veterinary care. The official municipal veterinarians detected most non-compliances when the following factors were present: an inspection was performed, the case was assessed using the information from the police or an animal shelter, or a complaint was received concerning insufficient veterinary care. The detection of violence against animals often resulted in a request for a police investigation. However, detecting violence appears to be a challenge. Research with a larger data set is needed to achieve a better understanding of the features and prevalence of violence against animals.

## Data Availability Statement

The raw data supporting the conclusions of this article will be made available by the authors, without undue reservation.

## Ethics Statement

The studies involving human participants were reviewed and approved by Ethical Review Board in the Humanities and Social and Behavioral Sciences, University of Helsinki. Written informed consent for participation was not required for this study in accordance with the national legislation and the institutional requirements.

## Author Contributions

EV contributed to the study design, the data collection, the analyses, and the preparation of the manuscript. TK, LH, and AV contributed to the study design, interpreting the results, and the manuscript preparation. All authors have read and approved the final manuscript.

## Funding

The study was funded by the Finnish Foundation of Veterinary Research, City of Helsinki, and the Doctoral School in Health Sciences, University of Helsinki.

## Conflict of Interest

EV works as an official veterinarian in the Environment Services of the City of Helsinki, and she was on duty during part of the study period. The remaining authors declare that the research was conducted in the absence of any commercial or financial relationships that could be construed as a potential conflict of interest.

## Publisher's Note

All claims expressed in this article are solely those of the authors and do not necessarily represent those of their affiliated organizations, or those of the publisher, the editors and the reviewers. Any product that may be evaluated in this article, or claim that may be made by its manufacturer, is not guaranteed or endorsed by the publisher.

## Footnotes

^1^Animal Welfare Act 247/1996. Available online at: https://www.finlex.fi/en/laki/kaannokset/1996/en19960247_20061430.pdf [Accessed August 26, 2021].

^2^Animal Welfare Decree 396/1996 Available online at: https://www.finlex.fi/en/laki/kaannokset/1996/en19960396_20060401.pdf [Accessed August 26, 2021].

^3^Valtioneuvoston asetus koirien, kissojen ja muiden pienikokoisten seura- ja harrastuseläinten suojelusta 592/2010 (Unofficial Translation: Decree of the Council of State 674/2010 Concerning the Protection of Dogs, Cats and Other Small-sized Companion and Hobby Animals). Available online at: https://www.finlex.fi/fi/laki/alkup/2010/20100674 [Accessed August 26, 2021].

^4^Administrative Procedure Act 434/2003. Available online at: https://www.finlex.fi/en/laki/kaannokset/2003/en20030434.pdf [Accessed August 26, 2021].

^5^Helsingin suunnitelma eläinten terveyden ja hyvinvoinnin valvonnasta sekä kunnan järjestämistä eläinlääkäripalveluista 2020–2024 (Translation: The Plan for Animal Health and Welfare Supervision and Organization of the Municipality Veterinary Services 2020-2024 in the City of Helsinki). City of Helsinki. Available online at: https://www.hel.fi/static/ymk/lomakkeet/eho-suunnitelma.pdf [Accessed August 26, 2021].

^6^Nature Conservation Act 1096/1996. Available online at: https://www.finlex.fi/fi/laki/kaannokset/1996/en19961096.pdf [Accessed August 26, 2021].
