# Supplementary material for: Animal Welfare Control—Inspection Findings and the Threshold for Requesting a Police Investigation
Source: Front Vet Sci. 2021 Sep 23;8:736084. doi: 10.3389/fvets.2021.736084 (PMC8495059; doi:10.3389/fvets.2021.736084)
Supplement: Supplementary file 1 [file Table_1.docx]

| Supplementary Material 1. The results of the univariable analysis (chi-square test and Fisher’s exact test) in cases of investigated animal welfare complaints. Summary of explanatory variables and their association with the outcome; official veterinarians’ detection of any non-compliances vs. no detection of non-compliances. Non-compliances were detected in 66.1% (460/696) of all cases. N = 696. | | | | | |
| --- | --- | --- | --- | --- | --- |
| Variable | Category | N | Cases with any detected non-compliances | % | P-value |
| **Species reported in the complaint** |  |  |  |  |  |
| Dog |  |  |  |  | 0.03 |
|  | Yes | 470 | 298 | 63 |  |
|  | No | 226 | 162 | 72 |  |
| Cat |  |  |  |  | 0.02 |
|  | Yes | 206 | 150 | 73 |  |
|  | No | 490 | 310 | 63 |  |
| Other companion animal |  |  |  |  | 0.1 |
|  | Yes | 72 | 54 | 75 |  |
|  | No | 624 | 406 | 65 |  |
| Several species |  |  |  |  | 0.1 |
|  | Yes | 53 | 41 | 77 |  |
|  | No | 643 | 419 | 65 |  |
| **Non-compliance type reported in the complaint** |  |  |  |  | < 0.001 |
|  | Insufficient basic maintenance and care only | 310 | 205 | 66 |  |
|  | Owner hospitalized, arrested, or deceased, or animal disturbing the environment | 66 | 28 | 42 |  |
|  | Insufficient veterinary care | 131 | 99 | 76 |  |
|  | Abuse/violence | 95 | 53 | 56 |  |
|  | Abandoned/overall neglect | 94 | 75 | 80 |  |
| **Source of complaint** |  |  |  |  | < 0.001 |
|  | Complaint from a member of the public/other stakeholder | 497 | 313 | 63 |  |
|  | Removal of the animals from their premises by the police | 199 | 147 | 74 |  |
| **Investigation** |  |  |  |  |  |
| Method of  investigation |  |  |  |  | < 0.001 |
|  | Investigated by phone or e-mail only | 113 | 22 | 20 |  |
|  | Assessed by the information received from a shelter or the police | 172 | 124 | 72 |  |
|  | Inspection(s) performed with prior warning | 74 | 52 | 70 |  |
|  | Inspection(s) performed without prior warning | 337 | 262 | 78 |  |
| More than one inspection/ police measure |  |  |  |  | < 0.001 |
|  | Yes | 118 | 100 | 85 |  |
|  | No | 578 | 360 | 62 |  |
| **Observed animals** |  |  |  |  |  |
| Several species detected |  |  |  |  | 0.005 |
|  | Yes | 80 | 64 | 80 |  |
|  | No | 616 | 396 | 64 |  |
|  |  |  |  |  |  |
|  |  |  |  |  |  |

| Supplementary Material 2. The results of the univariable analysis (chi-square test and Fisher's exact test or Fisher-Freeman-Halton exact test) in cases of detected non-compliance with the animal welfare legislation. Summary of explanatory variables and their association with the outcome; investigation request submitted to the police by official veterinarians vs. no investigation request submitted. An investigation request was made in 9.6% (44/460) of all cases with detected non-compliances. N = 460. | | | | | |
| --- | --- | --- | --- | --- | --- |
| Variable | Category | N | Cases leading to an investigation request to the police | % | P-value |
| **Species detected** |  |  |  |  |  |
| Dog |  |  |  |  | 0.1 |
|  | Yes | 304 | 34 | 11 |  |
|  | No | 156 | 10 | 6 |  |
| Cat |  |  |  |  | 0.1 |
|  | Yes | 161 | 10 | 6 |  |
|  | No | 299 | 34 | 11 |  |
| Other species |  |  |  |  | 1 |
|  | Yes | 64 | 6 | 9 |  |
|  | No | 396 | 38 | 10 |  |
| Several species |  |  |  |  | 0.5 |
|  | Yes | 64 | 4 | 6 |  |
|  | No | 396 | 40 | 10 |  |
| **Non-compliance type detected** |  |  |  |  | < 0.001 |
|  | Insufficient basic maintenance and care only | 190 | 11 | 6 |  |
|  | Insufficient veterinary care | 159 | 14 | 9 |  |
|  | Abuse/ violence | 22 | 9 | 41 |  |
|  | Abandoned/ overall neglect | 89 | 10 | 11 |  |
| **Investigation** |  |  |  |  |  |
| Source of complaint |  |  |  |  | 0.3 |
|  | Complaint from private source, at least one inspection performed without advance warning | 313 | 27 | 9 |  |
|  | Animal removed from premises by the police at least once | 147 | 17 | 12 |  |
| Method of investigation |  |  |  |  | 0.2 |
|  | Investigated by phone or e-mail only | 22 | 2 | 9 |  |
|  | Assessed by the information received from a shelter or the police | 124 | 15 | 12 |  |
|  | Inspection(s) performed with prior warning | 52 | 1 | 2 |  |
|  | Inspection(s) performed without prior warning | 262 | 26 | 10 |  |
| Executed measures |  |  |  |  | 0.004 |
|  | No measures or only advice | 162 | 9 | 6 |  |
|  | Orders/prohibitions | 118 | 7 | 6 |  |
|  | Urgent measures, temporary | 62 | 7 | 11 |  |
|  | Urgent measures, permanent | 118 | 21 | 18 |  |
| More than one inspection/ police measure |  |  |  |  | 0.003 |
|  | Yes | 100 | 18 | 20 |  |
|  | No | 360 | 26 | 7 |  |
|  |  |  |  |  |  |
|  |  |  |  |  |  |
